# A network meta-analysis of short-term efficacy of different single-drug targeted therapies in the treatment of renal cell carcinoma

**DOI:** 10.1042/BSR20170827

**Published:** 2017-12-07

**Authors:** Hong-Ling He, Wan-Xia Yao

**Affiliations:** 1 Academic Journals Publishing Center of Education Department, the First Affiliated Hospital of Xi’an Jiaotong University, Xi’an 710061, P.R. China; 2 School of Medicine, Xi’an Peihua University, Xi’an 710125, P.R. China

**Keywords:** Network meta-analysis, Renal cell carcinoma, Randomized controlled trial, Single-drug targeted therapy, Short-term efficacy

## Abstract

The network meta-analysis was conducted to compare the short-term efficacy of different single-drug targeted therapies in the treatment of renal cell carcinoma (RCC). We initially searched databases for randomized controlled trials (RCTs) on different single-drug targeted therapies in treating RCC. The meta-analysis combined the direct and indirect evidence to calculate the pooled odds ratios (OR) and draw surface under the cumulative ranking curves (SUCRA). A total of 14 eligible RCTs were ultimately selected. The partial response (PR) of Cabozantinib in the treatment of RCC was better than Sunitinib (OR = 2.7, 95%CI = 1.0–7.8), Everolimus (OR = 8.1, 95%CI = 3.1–25.0), and Temsirolimus (OR = 4.8, 95%CI = 1.0–31.0); the overall response rate (ORR) of Cabozantinib was better than Sorafenib, Sunitinib, Everolimus, and Temsirolimus (OR = 5.5, 95%CI = 1.1–27.0; OR = 2.6, 95%CI = 1.1–6.6; OR = 8.3, 95%CI = 3.5–20.0; OR = 5.7, 95%CI = 1.3–28.0 respectively). In addition, as for complete response (CR), PR, stable disease (SD), progressive disease (PD), ORR, and disease control rate (DCR), Cabozantinib had the best short-term efficacy among nine single-drug targeted therapies in the treatment of RCC (CR: 50.3%; PR: 93.6%; SD: 75.1%; PD: 68.0%; ORR: 95.5%; DCR: 73.2%); while Everolimus had the worst short-term efficacy (CR: 33.6%; PR: 22.3%; SD: 78.0%; PD: 35.9%; ORR: 22.9%; DCR: 19.9%). Our network meta-analysis indicated that Cabozantinib might have better short-term efficacy than other regimens in the treatment of RCC, while Everolimus might have poor short-term efficacy.

## Introduction

Malignant renal cell carcinoma (RCC) accounts for 2–3% of cancer incidence and results in more than 100,000 deaths worldwide every year [1]. It has historically been refractory to cytotoxic and hormonal agents [1]. In 2015, the estimated number of new cases of RCC in the United States was 61,560, representing approximately 3.7% of all new cases, and the estimated deaths were 14,080, accounting for nearly 2.4% of the total cancer mortality [2].

Surgery is curative in the majority of patients with local diseases. Nevertheless, local recurrence or distant metastasis occurs in up to 40% of patients treated for localized tumors and 5-year survival is less than 10% in this subgroup RCC, which accounts for 80–90% of kidney cancers and 70–80% of these are clear cell RCC [3,4]. Historically, tumors have been used to treat with cytokines with modest response rates (RR) and small survival benefit [5]. High-dose interleukin-2 remains an option for severe patients and is associated with durable remission in a small minority of patients [6,7]. Mutations in the Von Hippel–Lindau (VHL) gene are presented in most cases of sporadic RCC [8]. When VHL is inactivated, there is an up-regulation of hypoxia-inducible factors (HIFs) and subsequent activation of pathways involved with metabolism, inflammation, and angiogenesis [9]. This rationale has provided a theoretical basis for the development of several agents targeting angiogenesis, including vascular endothelial growth factor (VEGF) and mammalian target of rapamycin (mTOR) [10].

Since 2005, the U.S. Food and Drug Administration (FDA) and European Medicines Agency (EMA) have approved novel agents targeting the VEGF-pathway for patients with metastatic renal cell carcinoma (mRCC) based on large and well-powered randomized clinical trials. Motzer et al*.* [11,12] has reported that Sunitinib (an oral VEGF tyrosine kinase inhibitors) improved progression-free survival (PFS) compared with interferon-α. Two studies evaluated the role of bevacizumab (an intravenous antibody against VEGF) in first-line treatment of mRCC: Rini et al. [13,14] reported an improvement in PFS and a trend toward better overall survival (OS) in patients treated with bevacizumab plus interferon-α compared with interferon-α alone. While Escudier et al. (AVOREN trial) [15,16] corroborated the results for PFS in the arm treated with both drugs. Cabozantinib (XL184), an orally bioavailable tyrosine kinase inhibitor (TKI), targets multiple receptor tyrosine kinases, including VEGFRs [17]. Amzal et al*.* [18] proved that Cabozantinib was superior to all its comparators with a higher probability of longer PFS and OS in treatment of advanced RCC. Additionally, Motzer et al*.* [19] showed noninferiority of Pazopanib (another oral inhibitor of VEGF tyrosine kinase) to Sunitinib in terms of PFS.

The treatment of RCC has dramatically changed in the past several years with the approval of several new drugs since 2006 [20]. However, there is a few data to help choose the most effective drug which could improve patients’ outcomes, and predictive biomarkers of treatment response are also lacking [21]. Therefore, we aim to conduct a network meta-analysis in the first-line treatment of RCC in order to establish the most effective therapy in this setting.

## Material and methods

### Search strategy

Computer-based retrieval of PubMed and Cochrane Library databases (from inception to September 2016), combined with manual retrieval of related references were performed. Combining the keywords and free words, the search terms were showed as follows: Renal cell carcinoma (RCC), Targeted drugs, Sorafenib, Sunitini, Axitinib, Everolimus, Temsirolimus, Tivozanib, Dovitinib, Pazopanib, Cabozantinib etc.

### Inclusion and exclusion criteria

The inclusion criteria were as follows: (1) study design must be randomized controlled trials (RCTs); (2) studies investigated the efficacy of Sorafenib, Sunitinib, Everolimus, Temsirolimus, Axitinib, Tivozanib, Dovitinib, Pazopanib, and Cabozantinib in the treatment of RCC; (3) study subjects should be patients with RCC aging from 17 to 89 years; (4) the end outcomes of studies should include complete response (CR), partial response (PR), overall response rate (ORR), progressive disease (PD), stable disease (SD), and disease control rate (DCR). The exclusion criteria were as follows: (1) incomplete literature data; (2) non-RCTs; (3) duplications; (4) conference report, systematic review, and abstracts; (5) non-English literatures; (6) studies unrelated to RCC; (7) nonhuman studies; (8) studies unrelated to single-drug targeted therapy were excluded.

### Data extraction and quality assessment

Data were extracted by two researchers from the enrolled studies using a specifically designed form. Additionally, at least a third researcher should be consulted if agreement could not be reached between these two researchers. We had at least two researchers reviewed the RCTs according to Cochrane risk of bias assessment tools [22], which the following six domains were included: adequate sequence generation, allocation concealment, blinding, incomplete outcome data addressed, attrition and exclusions, free of selective reporting and free of other biases. Assigning a judgment of “yes,” “no,” or “unclear” for each domain is included in the assessment to designate a low, high, or unclear risk of bias respectively. The study was classified as having a low risk of bias if one or no domain was judged “unclear” or “no”. The study was classified as having a high risk of bias if four or more domains were deemed “unclear” or “no”. The study was classified as having a moderate risk of bias if two or three domains were deemed “unclear” or “no,” [23]. Review Manager 5 (RevMan 5.2.3, Cochrane Collaboration, Oxford, U.K.) was used to carry out the quality assessment and investigation of publication bias.

### Statistical analysis

First, traditional pairwise meta-analyses for studies that directly compared different treatment arms were performed. The pooled estimates of odd ratios (ORs) and 95% credible intervals (CrIs) of RCC were shown. We used Chi-square test and *I*-square test for testing heterogeneity among the studies [24]. Second, we used R 3.2.1 software to draw the network meta diagram, in which each node represented a variety of interventions, the node size represented the sample size, and the thickness of lines between the nodes represented the numbers of included researches. Third, Bayesian network meta-analyses were performed to compare different interventions to each other. Each analysis was on the basis of noninformative priors for effect sizes and precision. We checked and confirmed the convergence and lack of autocorrelation after four chains and a 20,000-simulation burn-in phase; we finally derived direct probability statements from an additional 50,000-simulation phase [25]. We used the node-splitting method to calculate the consistency of the model, which separated evidence on a particular comparison into direct and indirect evidence. [26]. For assisting in the interpretation of ORs, we calculated the probability of each intervention being the most effective or safest treatment method on the basis of a Bayesian approach using probability values summarized as surface under the cumulative ranking curve (SUCRA), the larger the SUCRA value, the better the rank of the intervention [27,28]. All computations were conducted by R (V.3.2.1) package gemtc (V.0.6) as well as the Markov Chain Monte Carlo engine Open BUGS (V.3.4.0).

## Results

### Baseline characteristics of included studies

**Figure 1:**
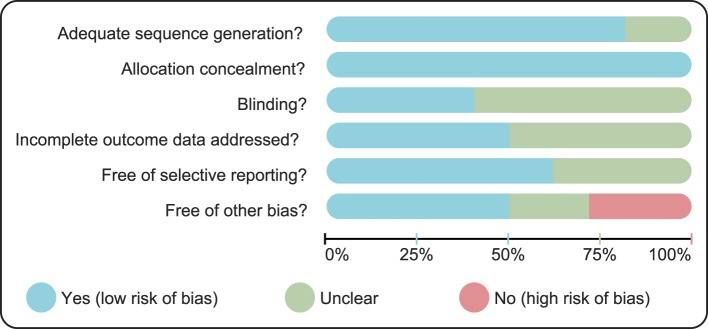
The Cochrane risk of bias assessment of nine single-drug targeted therapies in the treatment of RCC Abbreviation: RCC, renal cell carcinoma.

A total of 2456 publications were initially retrieved in the present study, including 2438 from keywords search, 18 from manual retrieve. Among of these studies, 8 for duplications, 625 for letters or reviews, 356 for nonhuman studies, and 372 for non-English literatures were eliminated. Moreover, 309 for non-RCTs, 358 articles unrelated to RCC, 412 articles unrelated to single-drug targeted therapy, 2 articles without data or incomplete data were also rejected from the rest of 1095 essays. Finally, 14 RCTs met the inclusion criteria and were selected into our meta-analysis from 2009 to 2017 [29–42] (Supplementary Figure S1). These studies included 12,047 patients with RCC and the number of patients treated with Sorafenib and Sunitinib was relatively larger. Among the 14 enrolled studies, 12 RCTs were Caucasians, and the rest RCT were Asians. Furthermore, 14 included studies were all two-arm trials. The baseline characteristics of included studies were summarized in Supplementary Table S1 and the Cochrane risk of bias assessment is shown in Figure 1.

### Pairwise meta-analysis of the short-term efficacy of nine single-drug targeted therapies

We evaluated the short-term efficacy of the nine single-drug targeted therapies using pairwise meta-analysis, and the results revealed that the PR of Sorafenib for the treatment of RCC performed worse than Axitinib and Tivozanib (OR = 0.41, 95%CI = 0.28–0.58; OR = 0.62, 95%CI = 0.42–0.92 respectively); the PR of Everolimus presented worse efficacy than Sunitinib and Cabozantinib (OR = 4.14, 95%CI = 2.36–7.28, OR = 5.56, 95%CI = 2.56–11.11 respectively); the SD of Sorafenib was relatively more effective than Tivozanib (OR = 1.75, 95%CI = 1.36–2.24); the SD of Everolimus was comparatively worse than Temsirolimus (OR = 0.27, 95%CI = 0.11–0.67); the PD of Everolimus worked worse than Temsirolimus (OR = 2.60, 95%CI = 1.00–6.74); the PD of Sorafenib was better than Tivozanib (OR = 0.52, 95%CI = 0.34–0.79); and the PD of Cabozantinib was better than that of Everolimus (OR = 2.31, 95%CI = 1.36–3.89). The ORR of Sunitinib was relatively more effective than Everolimus (OR = 4.18, 95%CI = 2.41–7.25); the ORR of Sorafenib worked worse than Axitinibe and Tivozanib (OR = 0.41, 95%CI = 0.28–0.58, OR = 0.61, 95%CI = 0.46–0.80 respectively); and the ORR of Cabozantinib was better than that of Everolimus and Sunitinib (OR = 0.18, 95%CI = 0.09–0.39, OR = 0.26, 95%CI = 0.13–0.54 respectively). The DCR of Sunitinib had better efficacy than Everolimus (OR = 1.92, 95%CI = 1.27–2.90); the DCR of Sorafenib presented worse than Axitinib (OR = 0.74, 95%CI = 0.57–0.96); while the DCR of Everolimus had worse efficacy than Temsirolimus (OR = 0.22, 95%CI = 0.09–0.58); and the DCR of Cabozantinib was better than that of Everolimus and Sunitinib (OR = 0.39, 95%CI = 0.24– 0.64, OR = 0.32, 95%CI = 0.16–0.64 respectively). However, all the regimens of CR had no obvious differences (Tables 1 and 2).

**Table 1:** Estimated OR and 95%CI from pairwise meta-analysis of efficacy events in renal cell carcinoma patients in terms of CR, PR, and SD

Included studies	Comparisons	Efficacy events		Pairwise meta-analysis		
		Treatment 1	Treatment 2	OR (95%CI)	*I*^2^	*P*_h_
**CR**						
1 study	A vs. B	1/20	1/29	1.47 (0.09–25.03)	NA	NA
1 study	A vs. D	1/253	1/259	1.02 (0.06-–.46)	NA	NA
2 studies	A vs. E	2/458	2/553	1.41 (0.20–10.10)	0.00%	0.727
1 study	A vs. F	2/257	3/260	0.67 (0.11–4.05)	NA	NA
1 study	A vs. G	1/286	1/284	0.99 (0.06–15.95)	NA	NA
1 study	B vs. C	3/233	1/238	3.09 (0.32–29.93)	NA	NA
1 study	C vs. D	1/59	1/31	0.52 (0.03–8.56)	NA	NA
1 study	B vs. I	1/78	1/79	1.01 (0.06–16.49)	NA	NA
2 studies	B vs. H	2/180	2/157	0.87 (0.12–6.27)	0.00%	0.845
1 study	C vs. I	1/188	1/187	0.99 (0.06–16.02)	NA	NA
**PR**						
1 study	A vs. B	1/20	2/29	0.71 (0.06–8.41)	NA	NA
1 study	A vs. D	19/253	20/259	0.97 (0.50–1.86)	NA	NA
2 studies	A vs. E	48/458	132/553	**0.41 (0.28–0.58)**	0.00%	0.641
1 study	A vs. F	58/257	83/260	**0.62 (0.42–0.92)**	NA	NA
1 study	A vs. G	11/286	11/284	0.99 (0.42–2.33)	NA	NA
1 study	B vs. C	59/233	18/238	**4.14 (2.36–7.28)**	NA	NA
1 study	C vs. D	6/59	5/31	0.59 (0.16–2.11)	NA	NA
1 study	B vs. I	13/78	35/79	0.25 (0.12–0.53)	NA	NA
2 studies	B vs. H	49/180	41/157	0.76 (0.35-1.66)	56.00%	0.132
1 study	I vs. C	40/187	9/188	**5.56 (2.56–11.11)**	NA	NA
**SD**						
1 study	A vs. B	13/20	18/29	1.13 (0.35–3.72)	NA	NA
1 study	A vs. D	153/253	157/259	0.99 (0.70–1.42)	NA	NA
2 studies	A vs. E	222/458	218/553	1.24 (0.96–1.61)	0.00%	0.601
2 studies	A vs. F	335/514	269/520	**1.75 (1.36–2.24)**	0.00%	0.899
1 study	A vs. G	149/286	147/284	1.01 (0.37–1.41)	NA	NA
1 study	B vs. C	121/233	137/238	0.80 (0.55–1.15)	NA	NA
1 study	C vs. D	16/59	18/31	**0.27 (0.11–0.67)**	NA	NA
1 study	B vs. I	28/78	26/79	1.14 (0.59–2.21)	NA	NA
2 studies	B vs. H	75/180	65/157	1.02 (0.48–2.17)	66.40%	0.085
1 study	C vs. I	116/188	116/187	0.99 (0.65–1.50)	NA	NA

Abbreviations: 95%CI, 95% confidence intervals; A, Sorafenib; B, Sunitinib; C, Everolimus; CR, complete response; D, Temsirolimus; E, Axitinib; F, Tivozanib; G, Dovitinib; H, Pazopanib; I, Cabozantinib; NA, not available; OR, odd ratios; PR, partial response; SD, stable disease.

**Table 2:** Estimated OR and 95%CI from pairwise meta-analysis of efficacy events in renal cell carcinoma patients in terms of PD, ORR, and DCR

Included studies	Comparisons	Efficacy events		Pairwise meta-analysis		
		Treatment 1	Treatment 2	OR (95%CI)	*I*^2^	*P*_h_
**PD**						
1 study	A vs. B	4/20	6/29	0.96 (0.23–3.95)	NA	NA
1 study	A vs. D	61/253	59/259	1.08 (0.72–1.62)	NA	NA
2 studies	A vs. E	88/458	98/553	1.01 (0.73–1.39)	0.00%	0.572
2 studies	A vs. F	37/514	68/520	**0.52 (0.34–0.79)**	0.00%	0.892
1 study	A vs. G	90/286	82/284	1.13 (0.79–1.62)	NA	NA
1 study	B vs. C	33/233	49/238	0.64 (0.39–1.03)	NA	NA
1 study	C vs. D	28/59	42978	**2.60 (1.00–6.74)**	NA	NA
1 study	B vs. I	20/78	14/79	1.60 (0.74–3.46)	NA	NA
2 studies	B vs. H	35/180	21/157	1.70 (0.15–19.49)	91.70%	0.001
1 study	C vs. I	51/188	26/187	**2.31 (1.36–3.89)**	NA	NA
**ORR**						
1 study	A vs. B	42755	47150	0.71 (0.06–8.41)	NA	NA
1 study	A vs. D	20/253	20/259	1.03 (0.54–1.96)	NA	NA
2 studies	A vs. E	48/458	132/553	**0.41 (0.28–0.58)**	0.00%	0.641
2 studies	A vs. F	119/514	172/520	**0.61 (0.46–0.80)**	0.00%	0.938
1 study	A vs. G	11/286	11/284	0.99 (0.42–2.33)	NA	NA
1 study	B vs. C	62/233	19/238	**4.18 (2.41–7.25)**	NA	NA
1 study	C vs. D	21732	42886	0.70 (0.20–2.42)	NA	NA
1 study	B vs. I	14/78	36/79	**0.26 (0.13–0.54)**	NA	NA
3 studies	B vs. H	2087/5741	238/864	0.94 (0.59–1.49)	57.70%	0.094
1 study	C vs. I	9/188	40/187	**0.18 (0.09–0.39)**	NA	NA
**DCR**						
1 study	A vs. B	14/20	20/29	1.05 (0.30–3.62)	NA	NA
1 study	A vs. D	183/253	177/259	1.21 (0.83–1.77)	NA	NA
2 studies	A vs. E	270/458	350/553	**0.74 (0.57–0.96)**	0.00%	0.465
2 studies	A vs. F	454/514	441/520	1.36 (0.95–1.94)	0.00%	0.775
1 study	A vs. G	160/286	158/284	1.01 (0.73–1.41)	NA	NA
1 study	B vs. C	183/233	156/238	**1.92 (1.27–2.90)**	NA	NA
1 study	C vs. D	23/59	23/31	**0.22 (0.09–0.58)**	NA	NA
1 study	B vs. I	42/78	62/79	**0.32 (0.16–0.64)**	NA	NA
2 studies	B vs. H	115/180	107/157	0.76 (0.15–3.75)	91.00%	0.001
1 study	C vs. I	125/188	156/187	**0.39 (0.24–0.64)**	NA	NA

Abbreviations: 95%CI, 95% confidence intervals; A, Sorafenib; B, Sunitinib; C, Everolimus; D, Temsirolimus; DCR, disease control rate; E, Axitinib; F, Tivozanib; G, Dovitinib; H, Pazopanib; I, Cabozantinib; NA, not available; OR, odd ratios; ORR, overall response rate; PD, progressive disease.

### Evidence network of the short-term efficacy of nine single-drug targeted therapies

**Figure 2:**
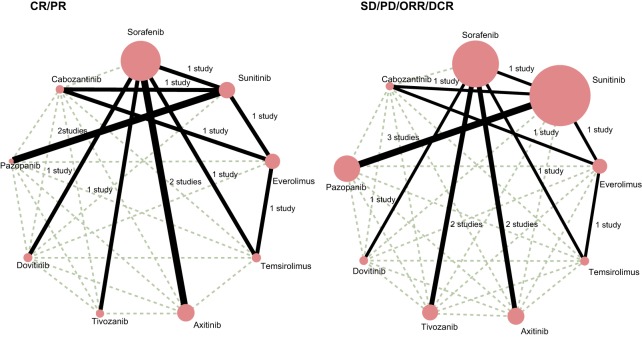
Network evidence plots of the CR, PR, SD, PD, ORR, and DCR of nine single-drug targeted therapies in the treatment of RCC Abbreviations: CR, complete response; DCR, disease control rate; H, Pazopanib; I, Cabozantinib; ORR, overall response rate; PD, progressive disease; PR, partial response; RCC, renal cell carcinoma; SD, stable disease.

Nine single-drug targeted therapies were included in our study: Sorafenib, Sunitinib, Everolimus, Temsirolimus, Axitini, Tivozanib, Dovitinib, Pazopanib, and Cabozantinib. In terms of CR, PR, ORR, PD, SD, and DCR, we observed that Sorafenib and Sunitinib obtained the largest number of patients in the treatment of RCC and more pairwise meta-analysis were made of Sorafenib vs. Axitinib, Sorafenib vs. Tivozanib as well as Sunitinib vs. Pazopanib based on the direct evidence (Figure 2).

### Inconsistency tests of the CR, PR, ORR, SD, PD, and DCR of nine single-drug targeted therapies

**Figure 3:**
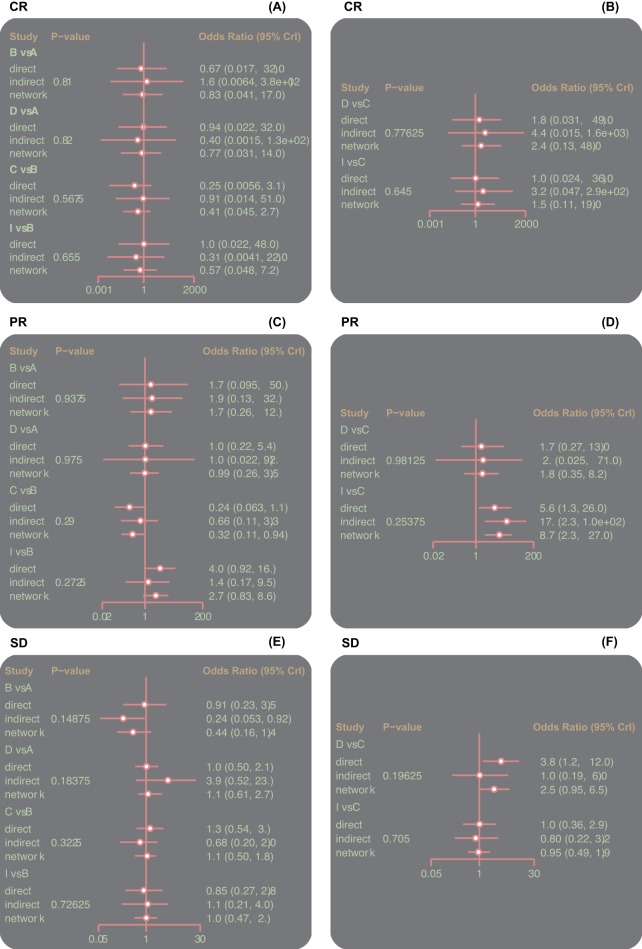
Inconsistent test plots of the CR, PR, and SD of nine single-drug targeted therapies in the treatment of RCC Abbreviations: A, Sorafenib; B, Sunitinib; C, Everolimus; CR, complete response; D, Temsirolimus; E, Axitinib; F, Tivozanib; G, Dovitinib; H, Pazopanib; I, Cabozantinib; PR, partial response; RCC, renal cell carcinoma; SD, stable disease.

**Figure 4:**
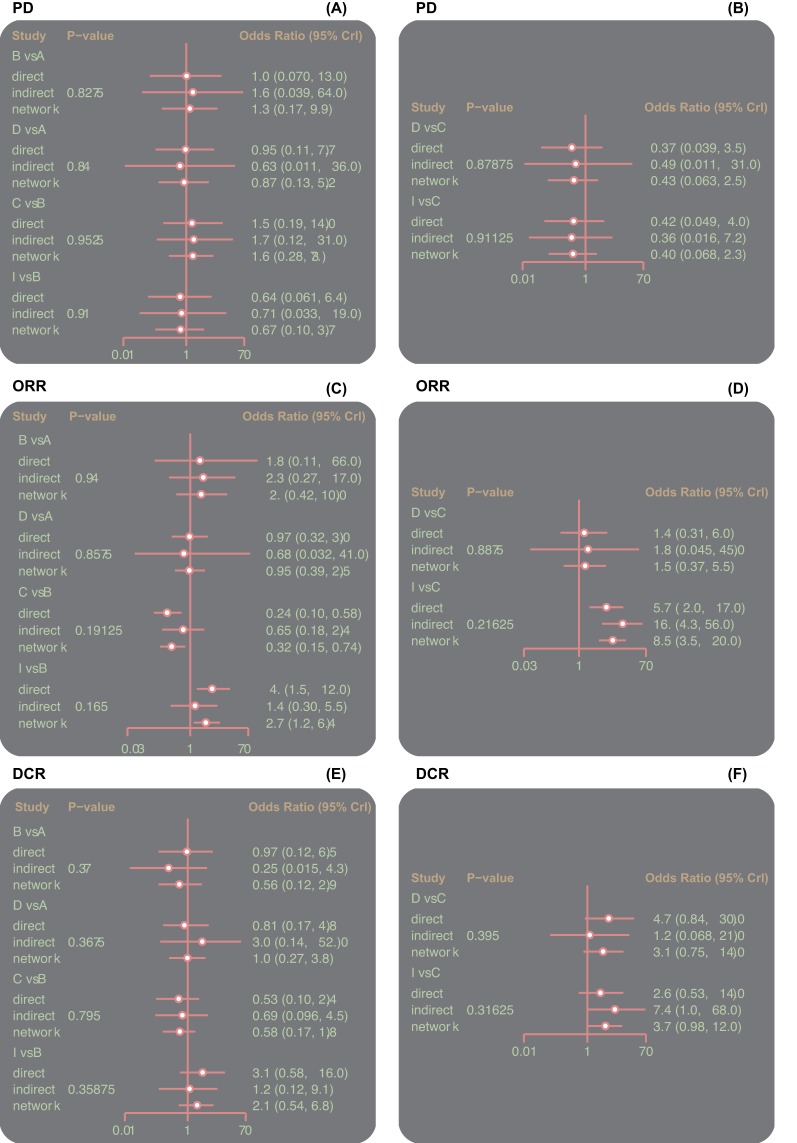
Inconsistent test plots of the PD, ORR, and DCR of nine single-drug targeted therapies in the treatment of RCC Abbreviations: A, Sorafenib; B, Sunitinib; C, Everolimus; D, Temsirolimus; DCR, disease control rate; E, Axitinib; F, Tivozanib; G, Dovitinib; H, Pazopanib; I, Cabozantinib; ORR, overall response rate; PD, progressive disease.

The ORR, CR, PR, SD, PD, and DCR outcomes were analyzed by inconsistency tests with the node-splitting method, and the analysis indicated that all outcomes of the direct and indirect evidence were consistent, thus the consistency model should be adopted (all *P*>0.05) (Figures 3 and 4).

### The results of network meta-analysis of the short-term efficacy of nine single-drug targeted therapies

The PR of Cabozantinib exhibited better efficacy than Sunitinib, Everolimus, and Temsirolimus (OR = 2.7, 95%CI = 1.0–7.8; OR = 8.1, 95%CI = 3.1–25.0; OR = 4.8, 95%CI = 1.0–31.0 respectively); the PR of Axitinib was better than that of Sorafenib (OR = 2.6, 95%CI = 1.1–6.1); the PR of Everolimus was comparatively worse than Sunitinib (OR = 0.33, 95%CI = 0.13–0.87); and the PR of Pazopanib had better efficacy than Everolimus (OR = 4.1, 95%CI = 1.1–15.0). The ORR of Cabozantinib was relatively more effective than Sorafenib, Sunitinib, Everolimus, and Temsirolimus (OR = 5.5, 95%CI = 1.1–27.0; OR = 2.6, 95%CI = 1.1–6.6; OR = 8.3, 95%CI = 3.5–20.0; OR = 5.7, 95%CI = 1.3–28.0 respectively); the ORR of Sorafenib worked worse than Axitinibe (OR = 2.5, 95%CI = 1.3–5.2); the ORR of Everolimus had worse effectiveness than that of Sunitinib (OR = 0.31, 95%CI = 0.15–0.70); and the ORR of Pazopanib was better than that of Everolimus (OR = 3.3, 95%CI = 1.3–8.5). Nevertheless, in terms of CR, SD, PD, and DCR, all the regimens in the treatment of RCC had no obvious difference (Tables 3 and 4).

**Table 3:** Odd ratios (OR) and 95% confidence intervals (95%CI) of nine drugs in the treatment of renal carcinoma in terms of CR, PR, and SD

OR (95% CI)								
**CR**								
**Sorafenib**	0.72 (0.038, 10.0)	0.26 (0.012, 6.4)	0.85 (0.049, 10.0)	0.65 (0.097, 9.4)	1.6 (0.18, 33.0)	0.64 (0.0014, 11.0)	0.84(0.030,24.0)	0.61 (0.015,18.0)
1.4 (0.099, 26.0)	**Sunitinib**	0.39 (0.059, 3.1)	1.2 (0.059,31.0)	1.1 (0.034,38.0)	2.6 (0.10, 1.4e+02)	0.88 (0.0018, 60.0)	1.1 (0.15, 12.0)	0.85 (0.081, 7.8)
3.8 (0.16, 80.0)	2.5 (0.32, 17.0)	**Everolimus**	2.6 (0.20, 47.0)	2.9 (0.047, 94.0)	6.0 (0.18, 4.5e+02)	2.5 (0.00076, 1.3e+02)	2.8 (0.16, 48.0)	1.9 (0.19, 25.0)
1.2 (0.098, 20.0)	0.81 (0.033, 17.0)	0.39 (0.021, 5.0)	**Temsirolimus**	0.89 (0.037,39.0)	2.5 (0.078, 61.0)	0.88 (0.00065, 42.0)	1.0 (0.032, 31.0)	0.66 (0.020, 26.0)
1.5 (0.11, 10.0)	0.92 (0.026, 29.0)	0.34 (0.011, 21.0)	1.1 (0.026, 27.0)	**Axitinib**	2.7 (0.095, 58.0)	0.93 (0.0035, 29.0)	1.3 (0.022, 41.0)	0.69 (0.012, 47.0)
0.62 (0.030, 5.7)	0.38 (0.0070, 9.7)	0.17 (0.0022, 5.5)	0.39 (0.016,13.0)	0.37 (0.017,11.0)	**Tivozanib**	0.42 (0.00011, 13.0)	0.43 (0.0058,17.0)	0.33 (0.0043, 14.0)
1.6 (0.091, 7.0e+02)	1.1 (0.017, 5.7e+02)	0.40 (0.0075, 1.3e+03)	1.1 (0.024, 1.5e+03)	1.1 (0.035, 2.8e+02)	2.4 (0.074, 9.1e+03)	**Dovitinib**	1.3 (0.014, 1.6e+03)	0.85 (0.0096, 1.0e+03)
1.2 (0.041, 34.0)	0.88 (0.083, 6.7)	0.36 (0.021, 6.2)	1.0 (0.032, 31.0)	0.78 (0.024, 45.0)	2.3 (0.060, 1.7e+02)	0.78 (0.00063,72.0)	**Pazopanib**	0.73 (0.027, 13.0)
1.7 (0.056, 66.0)	1.2 (0.13, 12.0)	0.52 (0.040, 5.2)	1.5 (0.038, 50.0)	1.5 (0.021,85.0)	3.1 (0.071, 2.3e+02)	1.2 (0.00096, 1.0e+02)	1.4 (0.075, 36.0)	**Cabozantinib**
**PR**								
**Sorafenib**	1.8 (0.38, 10.0)	0.61 (0.13, 3.5)	1.0 (0.32, 3.3)	**2.6 (1.1, 6.1)**	1.6 (0.51, 5.0)	1.0 (0.25, 4.3)	2.4 (0.39,19.0)	4.9 (0.92,36.0)
0.56 (0.097, 2.6)	**Sunitinib**	**0.33 (0.13, 0.87)**	0.57 (0.11, 2.5)	1.4 (0.22, 8.1)	0.89 (0.11, 6.0)	0.54 (0.055, 4.7)	1.3 (0.54, 3.2)	**2.7 (1.0, 7.8)**
1.6 (0.29, 7.7)	**3.1 (1.2, 7.9)**	**Everolimus**	1.7 (0.35, 6.6)	4.2 (0.69, 23.0)	2.6 (0.33, 18.0)	1.7 (0.16, 13.0)	**4.1 (1.1, 15.0)**	**8.1 (3.1, 25.0)**
0.97 (0.30, 3.1)	1.7 (0.40, 9.3)	0.58 (0.15, 2.9)	**Temsirolimus**	2.5 (0.59, 10.0)	1.5 (0.30, 8.3)	0.96 (0.16, 6.2)	2.3 (0.41,18.0)	**4.8 (1.0, 31.0)**
**0.38 (0.16, 0.89)**	0.71 (0.12, 4.6)	0.24 (0.043, 1.5)	0.39 (0.096, 1.7)	**Axitinib**	0.60 (0.15, 2.5)	0.39 (0.074, 2.1)	0.95 (0.13, 8.1)	1.9 (0.31,16.0)
0.63 (0.20, 2.0)	1.1 (0.17, 9.0)	0.39 (0.056, 3.0)	0.65 (0.12, 3.3)	1.7 (0.39, 6.6)	**Tivozanib**	0.63 (0.10, 4.4)	1.5 (0.18, 16.0)	3.1 (0.44, 31.0)
0.99 (0.23, 4.0)	1.8 (0.21, 18.0)	0.60 (0.077, 6.2)	1.0 (0.16, 6.2)	2.6 (0.48, 13.0)	1.6 (0.23, 9.9)	**Dovitinib**	2.5 (0.26, 29.0)	4.8 (0.56, 60.0)
0.41 (0.053, 2.6)	0.75 (0.31, 1.9)	**0.25 (0.065, 0.93)**	0.43 (0.057, 2.5)	1.1 (0.12, 7.9)	0.67 (0.062, 5.6)	0.40 (0.035, 3.9)	**Pazopanib**	2.0 (0.56, 9.0)
0.20 (0.028, 1.1)	**0.37 (0.13, 0.98)**	**0.12 (0.040, 0.32)**	**0.21 (0.033, 0.99)**	0.52 (0.063, 3.2)	0.32 (0.032, 2.3)	0.21 (0.017, 1.8)	0.50 (0.11, 1.8)	**Cabozantinib**
**SD**								
**Sorafenib**	0.42 (0.17, 1.4)	0.45 (0.17, 1.4)	1.1 (0.57, 2.8)	0.79 (0.42, 1.5)	0.57 (0.30, 1.0)	1.0 (0.44, 2.4)	0.40 (0.14, 1.6)	0.44 (0.15, 1.5)
2.4 (0.72, 5.9)	**Sunitinib**	1.1 (0.51, 1.9)	2.7 (0.85, 7.0)	1.9 (0.47, 5.6)	1.3 (0.35, 3.9)	2.3 (0.53, 7.7)	0.95 (0.50, 1.9)	1.0 (0.46, 2.1)
2.2 (0.72, 5.8)	0.94 (0.53, 1.9)	**Everolimus**	2.5 (0.92, 6.4)	1.8 (0.46, 5.6)	1.3 (0.34, 3.9)	2.2 (0.56, 7.7)	0.90 (0.39, 2.5)	0.96 (0.47, 2.1)
0.89 (0.36, 1.8)	0.37 (0.14, 1.2)	0.40 (0.16, 1.1)	**Temsirolimus**	0.71 (0.22, 1.7)	0.51 (0.15, 1.2)	0.88 (0.26, 2.5)	0.36 (0.11, 1.3)	0.38 (0.12, 1.2)
1.3 (0.69, 2.4)	0.54 (0.18, 2.1)	0.56 (0.18, 2.2)	1.4 (0.59, 4.5)	**Axitinib**	0.72 (0.30, 1.7)	1.2 (0.46, 3.6)	0.51 (0.14, 2.4)	0.56 (0.16, 2.3)
1.8 (0.96, 3.3)	0.74 (0.26, 2.9)	0.78 (0.26, 3.0)	2.0(0.84, 6.5)	1.4 (0.58, 3.3)	**Tivozanib**	1.8 (0.65, 5.5)	0.71 (0.21, 3.7)	0.77 (0.22, 3.2)
1.0 (0.42, 2.3)	0.43 (0.13, 1.9)	0.46 (0.13, 1.8)	1.1 (0.40, 3.8)	0.80 (0.28, 2.2)	0.57 (0.18, 1.5)	**Dovitinib**	0.42 (0.10, 2.1)	0.44 (0.11, 2.0)
2.5 (0.61, 7.2)	1.1 (0.54, 2.0)	1.1 (0.41, 2.6)	2.8 (0.75, 8.8)	2. (0.41, 7.1)	1.4 (0.27, 4.7)	2.4 (0.48, 9.6)	**Pazopanib**	1.1 (0.37, 2.6)
2.3 (0.65, 6.7)	0.99 (0.48, 2.2)	1.0 (0.47, 2.1)	2.6 (0.82, 8.2)	1.8 (0.44, 6.4)	1.3 (0.32, 4.5)	2.3 (0.50, 8.8)	0.94 (0.38, 2.7)	**Cabozantinib**

Notes: OR and 95%CI below the treatments should be read from row to column while above the treatments should be read from column to row. CR = complete response; PR = partial response; SD = stable disease.

**Table 4:** Odd ratios (OR) and 95% confidence intervals (95%CI) of nine drugs in the treatment of renal carcinoma in terms of PD, ORR, and DCR

OR (95% CI)								
**PD**								
**Sorafenib**	1.3 (0.15, 10.0)	2.1 (0.20, 18.0)	0.83 (0.13, 5.4)	3.5 (0.42, 27.0)	1.5 (0.31, 7.3)	0.87 (0.10, 7.9)	0.75 (0.052,11.0)	0.84 (0.066, 11.0)
0.78 (0.095, 6.6)	**Sunitinib**	1.6 (0.32, 8.7)	0.66 (0.076, 6.2)	2.7 (0.14, 60.0)	1.1 (0.080, 17.0)	0.70 (0.032,16.0)	0.60 (0.12, 2.9)	0.65 (0.11, 4.3)
0.48 (0.055, 4.9)	0.63 (0.12, 3.1)	**Everolimus**	0.41 (0.052, 3.2)	1.7 (0.087, 36.0)	0.69 (0.045,12.0)	0.42 (0.019, 11.0)	0.38 (0.039, 3.8)	0.41 (0.073, 2.5)
1.2 (0.19, 7.6)	1.5 (0.16, 13.0)	2.5 (0.31, 19.0)	**Temsirolimus**	4.3 (0.26,62.0)	1.7 (0.15, 21.0)	1.0 (0.066, 22.0)	0.91 (0.063, 14.0)	1.0 (0.088,13.0)
0.29 (0.037, 2.4)	0.37 (0.017, 7.2)	0.59 (0.027,12.0)	0.23 (0.016, 3.8)	**Axitinib**	0.42 (0.030, 6.2)	0.25 (0.013, 5.3)	0.23 (0.0058, 6.7)	0.24 (0.0093, 7.1)
0.68 (0.14, 3.3)	0.91 (0.058, 12.0)	1.4 (0.084, 22.0)	0.58 (0.048, 6.7)	2.4 (0.16,33.0)	**Tivozanib**	0.60 (0.048, 7.6)	0.52 (0.024,13.0)	0.60 (0.027, 12.0)
1.2 (0.13, 9.7)	1.4 (0.064, 31.0)	2.4 (0.088, 52.0)	0.97 (0.046, 15.0)	4.0 (0.19,75.0)	1.7 (0.13, 21.0)	**Dovitinib**	0.88 (0.025, 28.0)	0.97 (0.035, 29.0)
1.3 (0.093, 19.0)	1.7 (0.34, 8.3)	2.6 (0.27, 26.0)	1.1 (0.069, 16.0)	4.4 (0.15, 1.7e+02)	1.9 (0.078, 42.0)	1.1 (0.036, 41.0)	**Pazopanib**	1.1 (0.10,14.0)
1.2 (0.092, 15.0)	1.5 (0.23, 9.0)	2.4 (0.40, 14.0)	0.99 (0.078, 11.0)	4.1 (0.14, 1.1e+02)	1.7 (0.085, 37.0)	1.0 (0.034,29.0)	0.92 (0.073, 9.6)	**Cabozantinib**
**ORR**								
**Sorafenib**	2.0 (0.42, 9.4)	0.64 (0.15, 3.1)	0.93 (0.35, 2.5)	**2.5 (1.3, 5.2)**	1.7 (0.88, 3.1)	1.0 (0.30, 3.3)	2.1 (0.41, 11.0)	**5.5 (1.1, 27.0)**
0.49 (0.11, 2.4)	**Sunitinib**	**0.31 (0.15, 0.70)**	0.46 (0.11, 1.9)	1.2 (0.22, 6.3)	0.81 (0.15, 4.6)	0.49 (0.070, 3.8)	1.0 (0.64, 2.0)	**2.6 (1.1, 6.6)**
1.6 (0.33, 6.8)	**3.2 (1.4, 6.7)**	**Everolimus**	1.5 (0.34, 5.4)	4. (0.71, 18.0)	2.6 (0.47, 13.0)	1.6 (0.22, 11.0)	**3.3 (1.3, 8.5)**	**8.3 (3.5, 20.0)**
1.1 (0.40, 2.9)	2.2 (0.53, 9.5)	0.69 (0.18, 2.9)	**Temsirolimus**	2.7 (0.80, 9.2)	1.8 (0.53, 5.6)	1.1 (0.22, 5.0)	2.2 (0.52,12.0)	**5.7 (1.3, 28.0)**
**0.40 (0.19, 0.79)**	0.80 (0.16, 4.6)	0.25 (0.055, 1.4)	0.36 (0.11, 1.3)	**Axitinib**	0.66 (0.26, 1.7)	0.40 (0.10, 1.6)	0.84 (0.15, 5.5)	2.1 (0.38, 13.0)
0.61 (0.32, 1.1)	1.2 (0.22, 6.9)	0.39 (0.079, 2.1)	0.56 (0.18, 1.9)	1.5 (0.60, 3.9)	**Tivozanib**	0.61 (0.16, 2.3)	1.3 (0.22, 7.9)	3.3 (0.57,19.0)
0.99 (0.30, 3.3)	2.0 (0.27, 14.0)	0.64 (0.090, 4.5)	0.91 (0.20, 4.6)	2.5 (0.62, 9.7)	1.6 (0.44, 6.4)	**Dovitinib**	2.1 (0.26, 17.0)	5.4 (0.69, 41.0)
0.48 (0.087, 2.4)	0.97 (0.51, 1.6)	**0.30 (0.12, 0.78)**	0.45 (0.084, 1.9)	1.2 (0.18, 6.6)	0.78 (0.13, 4.6)	0.47 (0.060, 3.8)	**Pazopanib**	2.5 (0.90, 6.6)
**0.18 (0.038, 0.93)**	**0.38 (0.15, 0.91)**	**0.12 (0.050, 0.29)**	**0.17 (0.036, 0.77)**	0.47 (0.079, 2.6)	0.30 (0.054, 1.8)	0.19 (0.024, 1.4)	0.40 (0.15, 1.1)	**Cabozantinib**
**DCR**								
**Sorafenib**	0.59 (0.13, 2.8)	0.34 (0.070, 1.7)	1.0 (0.29, 4.3)	1.4 (0.47, 3.9)	0.73 (0.25, 2.3)	0.99 (0.23, 4.3)	0.76 (0.13, 5.4)	1.2 (0.22, 7.6)
1.7 (0.36, 7.6)	**Sunitinib**	0.57 (0.19, 1.8)	1.8 (0.37, 9.4)	2.4 (0.34, 15.0)	1.3 (0.19, 8.5)	1.7 (0.20, 13.0)	1.3 (0.47,4.0)	2.1 (0.59, 7.6)
3.0 (0.60, 14.0)	1.7 (0.55, 5.4)	**Everolimus**	3.1 (0.72, 14.0)	4.2 (0.61,30.0)	2.2 (0.32, 16.0)	3.0(0.34, 23.0)	2.3 (0.48,11.0)	3.7 (1.1,13.0)
0.96 (0.23, 3.5)	0.56 (0.11, 2.7)	0.32 (0.070, 1.4)	**Temsirolimus**	1.4 (0.22, 7.6)	0.71 (0.12, 4.1)	0.96 (0.12, 6.9)	0.74 (0.10, 5.3)	1.2 (0.19, 6.5)
0.71 (0.26, 2.1)	0.41 (0.065, 2.9)	0.24 (0.033, 1.6)	0.74 (0.13, 4.6)	**Axitinib**	0.53 (0.12, 2.6)	0.70 (0.11, 4.2)	0.55 (0.069, 5.2)	0.87 (0.11, 7.3)
1.4 (0.43, 3.9)	0.78 (0.12, 5.2)	0.45 (0.063, 3.1)	1.4 (0.24, 8.3)	1.9 (0.38, 8.3)	**Tivozanib**	1.3 (0.21, 8.5)	1.0 (0.12, 9.8)	1.7 (0.21, 13.0)
1.0 (0.23, 4.3)	0.59 (0.078, 4.9)	0.34 (0.043, 2.9)	1.0 (0.14, 8.4)	1.4 (0.24, 8.7)	0.75 (0.12, 4.9)	**Dovitinib**	0.79 (0.080, 8.7)	1.3 (0.13, 13.0)
1.3 (0.19, 8.0)	0.77 (0.25, 2.1)	0.44 (0.091, 2.1)	1.4 (0.19, 9.8)	1.8 (0.19, 15.0)	0.96 (0.10, 8.2)	1.3 (0.11,13.0)	**Pazopanib**	1.6 (0.29, 8.5)
0.80 (0.13, 4.6)	0.48 (0.13, 1.7)	0.27 (0.079, 0.93)	0.84 (0.15, 5.3)	1.1 (0.14, 9.4)	0.60 (0.077, 4.8)	0.80 (0.077, 7.8)	0.63 (0.12, 3.5)	**Cabozantinib**

Notes: OR and 95%CI below the treatments should be read from row to column while above the treatments should be read from column to row. Abbreviations: DCR, disease control rate; ORR, overall response rate; PD, progressive disease.

### Cumulative probability of the short-term efficacy of nine single-drug targeted therapies

As shown in Table 5, the SUCRA values of the efficacy of the nine targeted therapy regimens demonstrated that PR and ORR of RCC patients treated with Cabozantinib ranked the highest (PR: 93.6%; ORR: 95.5%); SD and PD of RCC patients treated with Pazopanib ranked the highest (SD: 78.0%; PD: 70.3%); CR of patients treated by Tivozanib ranked the highest (74.2%); the DCR of RCC patients treated with Axitinib ranked the highest (78.4%). However, the CR, PR, PD, ORR, and DCR of RCC patients treated with Everolimus exhibited relatively poor trend (CR: 33.6%; PR: 22.3%; PD: 35.9%; ORR: 22.9%; DCR: 19.9%).

**Table 5:** SUCRA values of nine treatment modalities under six endpoint outcomes

Treatments	SUCRA values					
	CR	PR	SD	PD	ORR	DCR
**A**	0.634	0.355	0.294	0.629	0.355	0.627
**B**	0.551	0.608	0.760	0.519	0.658	0.390
**C**	0.336	0.223	0.728	0.359	0.229	0.199
**D**	0.583	0.392	0.230	0.687	0.338	0.653
**E**	0.537	0.776	0.472	0.272	0.783	**0.784**
**F**	**0.742**	0.584	0.663	0.489	0.608	0.474
**G**	0.528	0.396	0.323	0.662	0.395	0.611
**H**	0.588	0.730	**0.780**	**0.703**	0.678	0.530
**I**	0.503	**0.936**	0.751	0.680	**0.955**	0.732

Abbreviations: A, Sorafenib; B, Sunitinib; C, Everolimus; CR, complete response; D, Temsirolimus; DCR, disease control rate; E, Axitinib; F, Tivozanib; G, Dovitinib; H, Pazopanib; I, Cabozantinib; ORR, overall response rate; PD, progressive disease; PR, partial response; SD, stable disease; SUCRA, surface under the cumulative ranking curves.

### Cluster analysis of the short-term efficacy of nine single-drug targeted therapies

**Figure 5:**
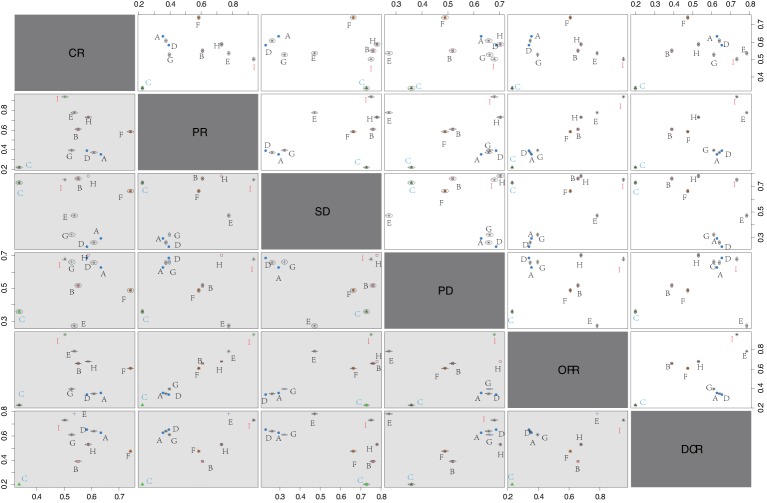
Cluster ranking plots of CR, PR, SD, PD, ORR, and DCR of nine single-drug targeted therapies in the treatment of RCC Abbreviations: A, Sorafenib; B, Sunitinib; C, Everolimus; CR, complete response; D, Temsirolimus; DCR, disease control rate; E, Axitinib; F, Tivozanib; G, Dovitinib; H, Pazopanib; I, Cabozantinib; ORR, overall response rate; PD, progressive disease; PR, partial response; RCC, renal cell carcinoma; SD, stable disease.

The cluster analysis revealed that CR, PR, SD, PD, ORR, and DCR of RCC patients had better effectiveness after treated with Cabozantinib (CR: 50.3%; PR: 93.6%; SD: 75.1%; PD: 68.0%; ORR: 95.5%; DCR: 73.2%), while received relatively poor results after treated with Everolimus (CR: 33.6%; PR: 22.3%; SD: 78.0%; PD: 35.9%; ORR: 22.9%; DCR: 19.9%) (Figure 5). Thus, Cabozantinib might be the best regimen in the treatment of RCC and Everolimus probably was the worst regimen in the treatment of RCC.

## Discussion

RCC is known as a cancer that results from a genetic inactivation of the VHL tumor suppressor gene leading to an up-regulation of VEGF. Targeted therapies against VEGF receptors have piqued substantial interest among clinicians and researchers, and these drugs are now the standard of care in the treatment of advanced RCC [43]. Our network meta-analysis included 14 RCTs and the direct results of our research revealed that Cabozantinib has been shown to be a superior chose among the nine regimens in the treatment of RCC, and Everolimus could be the inferior regimen when compared with other regimens in the treatment of RCC.

RCC is usually recognized as a highly vascular tumor, so several drugs affecting VEGF signaling have been approved for the treatment of metastatic disease, and the improved understanding of the biology of RCC has contributed to the identification of two cellular signaling pathways that are relevant for molecular-targeting therapy, which are the VEGF pathway and the mTOR pathway [44]. VEGF activity can be inhibited by bevacizumab, which is a monoclonal antibody that binds circulating VEGF and thereby prevents it from binding to its receptor. TKI including Axitinib, Sorafenib, Sunitinib, Pazopanib, and Tivozanib inhibit VEGF signaling by targeting the intracellular domain of the VEGFR; however, mTOR–raptor signaling is a potential target for antitumor therapy and mTOR inhibitors are currently under investigation for the treatment of various human cancers [44]. Cabozantinib represents a very good option for mRCC treatment, with outstanding outcomes in terms of response rate, PFS, OS, and quick time to treatment response [45]. Everolimus is an orally active mTOR inhibitor, and VEGF is a potent proangiogenic protein that plays an important role in tumor angiogenesis [10] and acts by binding to the VEGFR on endothelial cells [46]. The mTOR also interacts with rictor (rapamycin-insensitive companion of mTOR) and recent findings have suggested that the rapamycin-insensitive effect of mTOR on cell viability is up-regulated in many cancers. Thus, mTOR has dual rapamycin-sensitive (mTORC1) and rapamycin-insensitive (mTORC2) functions, indicating that treatment with inhibitors of the rapamycin-sensitive component (Temsirolimus and Everolimus) will not completely block mTOR activity [47,48]. Accordingly, these agents induce cell-cycle arrest and also inhibit tumor angiogenesis by reducing the synthesis of VEGF [49].

Furthermore, the results of SUCRA analysis provided further evidence that PR and ORR of Cabozantinib, SD and PD of Pazopanib, CR of Tivozanib and DCR of Axitinib ranked the highest and while the CR, PR, PD, ORR, and DCR of Everolimus ranked the lowest. A previous study has shown that targeted therapies act by blocking essential biochemical pathways or mutant proteins that are required for tumor cell growth and survival and the ability to inhibit the tumor cell growth and survival may explain the different efficacy of different single-drug targeted therapies for RCC [50]. Rossetti et al. [51] confirmed that Pazopanib was effective, even in reduced dosing, and well tolerated and suggested that Everolimus may represent an opportunity to continue a therapy when patients cannot further tolerate angiogenesis inhibitors or develop a resistance. Recently, two randomized Phase III trials (METEOR and CheckMate 025) demonstrated the inferiority of Everolimus in second-line setting compared with the TKI Cabozantinib and with the immune checkpoint inhibitor nivolumab respectively [52]. In Grassi’s study, the ORR was 21% with Cabozantinib as compared with 5% with Everolimus, and SD occurred as the best response in 62% of patients in each group, while progressive disease occurred in 14% of patients treated with Cabozantinib and 27% of patients treated with Everolimus [53]. Ruiz-Morales et al. [54] suggested that the potential of Cabozantinib will continue to be explored in patients with RCC in studies such as a phase II trial of Cabozantinib vs. Sunitinib in the first-line setting for RCC, which recently had a press release declaring a statistically significant improvement in PFS for Cabozantinib compared with Sunitinib in patients with intermediate-or poor-risk RCC, which were all consistent with our results.

Stated thus, our meta-analysis provides us strong evidence that Cabozantinib is a superior chose in the treatment of RCC. While the targeted agents could present specific toxicity profiles which differ from conventional chemotherapeutic agents [55]: potentially fatal gastrointestinal perforation associated with bevacizumab; hypothyroidism and cardiovascular toxicity caused by Everolimus and hypertension and cutaneous reactions (i.e. hand–foot syndrome) became the common adverse effects of antiangiogenic agents [56]. Therefore, the skillful management of adverse events should be made through further efforts.

## Supporting information

**Supplementary Table 1:** The baseline characteristics for included studies.

**Supplementary Figure 1:** Flowchart showing literature search and study selection, 14 clinical RTCs met the inclusion criteria were included in this network meta-analysis.

## Abbreviations

DCR: disease control rate
mRCC: metastatic renal cell carcinoma
mTOR: mammalian target of rapamycin
ORR: overall response rate
PD: progressive disease
PFS: progression-free survival
RCC: renal cell carcinoma
RCT: randomized controlled trial
TKI: tyrosine kinase inhibitor
VEGF: vascular endothelial growth factor
