# Supplementary material for: A network meta-analysis of short-term efficacy of different single-drug targeted therapies in the treatment of renal cell carcinoma
Source: Biosci Rep. 2017 Dec 7;37(6):BSR20170827. doi: 10.1042/BSR20170827 (PMC5719001; doi:10.1042/BSR20170827)
Supplement: Supplementary file 1 [file bsr20170827_Supp1.pdf]

Supplementary Table 1. The baseline characteristics for included studies.

First author	Year	Country	Ethnicity	Interventions		Total	Number		Age (years)	
				T1	T2		T1	T2	T1	T2
Patel SB	2016	USA	Caucasians	C	D	90	59	31	61.6(52.9~67.9)	59.6 (52.3~69.2)
Motzer RJ-a	2014	USA	Caucasians	A	G	570	286	284	62 (18~81)	61 (29~89)
Motzer RJ-b	2014	USA	Caucasians	B	C	471	233	238	62(29~84)	62(20~89)
Hutson TE	2014	USA	Caucasians	A	D	512	253	259	61(21~80)	60(19~82)
Wong HH	2013	UK	Caucasians	A	F	517	257	260	59 (23~85)	59 (23~83)
Ueda T	2013	Japan	Asians	A	E	723	362	361	61 (22~80)	61 (20~82)
Motzer RJ	2013	USA	Caucasians	A	F	517	257	260	59(23~85）	59（23~83)
Hutson TE	2013	USA	Caucasians	A	E	288	96	192	58·0 (20~77)	58·0 (23~83)
Dudek AZ	2009	USA	Caucasians	A	B	49	20	29	58.5 (39~69)	62 (32~75)
Choueiri TK	2017	USA	Caucasians	B	I	157	78	79	64(31-87)	63 (40-82)
Ruiz-Morales JM	2016	Canada	Caucasians	B	H	7438	6519	919	62(56-69)	65 (58-73)
Kim JH	2016	Korea	Asians	B	H	172	100	72	57(17-83)	60 (34-80)
Choueiri TK	2015	USA	Caucasians	C	I	375	188	187	NR	NR
Escudier B	2014	France	Caucasians	B	H	168	82	86	62	64

Notes: T = treatment; A = Sorafenib; B = Sunitinib; C = Everolimus; D = Temsirolimus; E = Axitinib; F = Tivozanib; G = Dovitinib; H = Pazopanib; I = Cabozantinib.

Supplementary Figure 1. Flowchart showing literature search and study selection, 14 clinical RTCs met the inclusion criteria were included in this network meta-analysis.

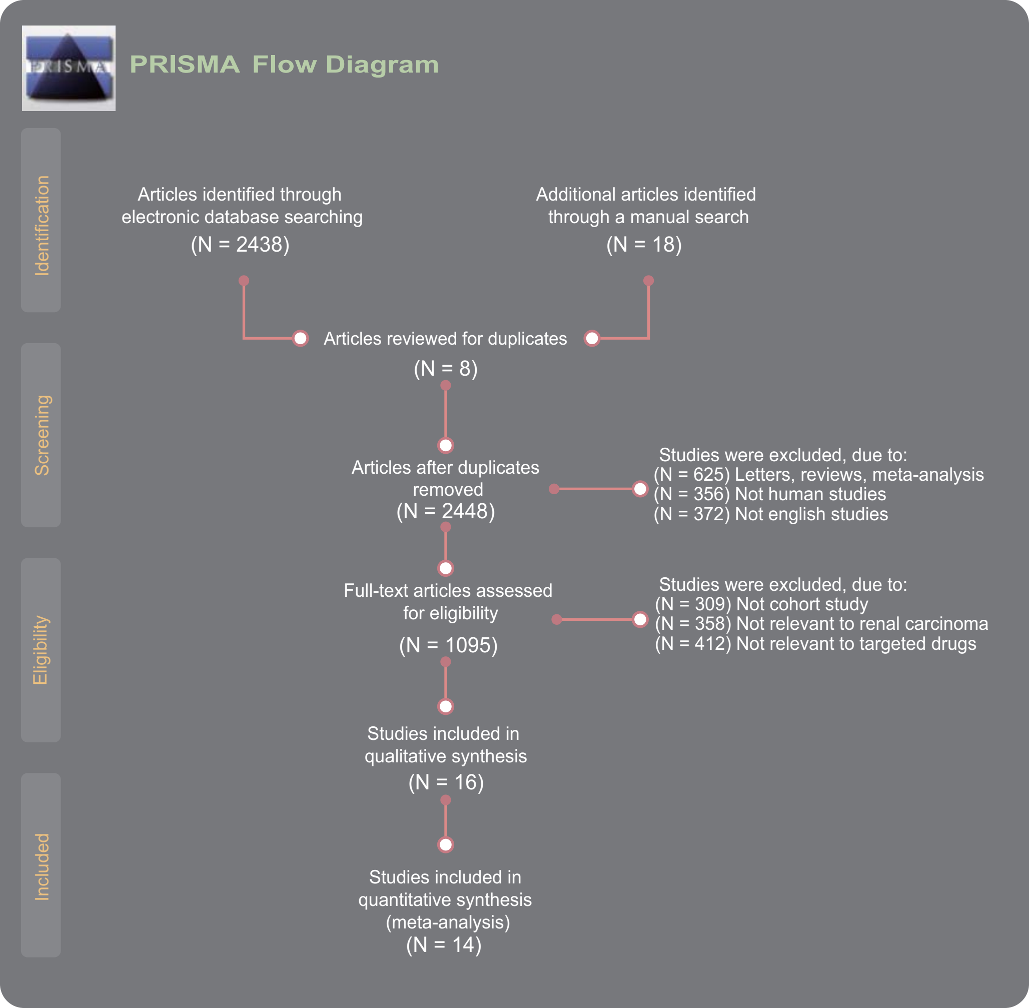
PRISMA Flow Diagram
Identification
Screening
Eligibility
Included
Articles identified through electronic database searching (N = 2438)
Additional articles identified through a manual search (N = 18)
Articles reviewed for duplicates (N = 8)
Articles after duplicates removed (N = 2448)
Studies were excluded, due to:
(N = 625) Letters, reviews, meta-analysis
(N = 356) Not human studies
(N = 372) Not english studies
Full-text articles assessed for eligibility (N = 1095)
Studies were excluded, due to:
(N = 309) Not cohort study
(N = 358) Not relevant to renal carcinoma
(N = 412) Not relevant to targeted drugs
Studies included in qualitative synthesis (N = 16)
Studies included in quantitative synthesis (meta-analysis) (N = 14)
